# Sex differences in inflammation in the hippocampus and amygdala across the lifespan in rats: associations with cognitive bias

**DOI:** 10.1186/s12979-022-00299-4

**Published:** 2022-10-06

**Authors:** Travis E. Hodges, Stephanie E. Lieblich, Rebecca K. Rechlin, Liisa A. M. Galea

**Affiliations:** 1 Department of Psychology, University of British Columbia, Vancouver, Canada; 2 Djavad Mowafaghian Centre for Brain Health, University of British Columbia, Vancouver, Canada; 3 Graduate Program in Neuroscience, University of British Columbia, Vancouver, Canada

**Keywords:** Adolescence, Young adult, Middle-age, TNF-α, IL-1β, Cognitive bias, Doublecortin, Dorsal hippocampus, Basolateral amygdala, Ventral hippocampus

## Abstract

**Background:**

Cognitive symptoms of major depressive disorder, such as negative cognitive bias, are more prevalent in women than in men. Cognitive bias involves pattern separation which requires hippocampal neurogenesis and is modulated by inflammation in the brain. Previously, we found sex differences in the activation of the amygdala and the hippocampus in response to negative cognitive bias in rats that varied with age. Given the association of cognitive bias to neurogenesis and inflammation, we examined associations between cognitive bias, neurogenesis in the hippocampus, and cytokine and chemokine levels in the ventral hippocampus (HPC) and basolateral amygdala (BLA) of male and female rats across the lifespan.

**Results:**

After cognitive bias testing, males had more IFN-γ, IL-1β, IL-4, IL-5, and IL-10 in the ventral HPC than females in adolescence. In young adulthood, females had more IFN-γ, IL-1β, IL-6, and IL-10 in the BLA than males. Middle-aged rats had more IL-13, TNF-α, and CXCL1 in both regions than younger groups. Adolescent male rats had higher hippocampal neurogenesis than adolescent females after cognitive bias testing and young rats that underwent cognitive bias testing had higher levels of hippocampal neurogenesis than controls. Neurogenesis in the dorsal hippocampus was negatively associated with negative cognitive bias in young adult males.

**Conclusions:**

Overall, the association between negative cognitive bias, hippocampal neurogenesis, and inflammation in the brain differs by age and sex. Hippocampal neurogenesis and inflammation may play greater role in the cognitive bias of young males compared to a greater role of BLA inflammation in adult females. These findings lay the groundwork for the discovery of sex-specific novel therapeutics that target region-specific inflammation in the brain and hippocampal neurogenesis.

**Supplementary Information:**

The online version contains supplementary material available at 10.1186/s12979-022-00299-4.

## Highlights

• Adolescent male rats had more hippocampal inflammation than females after cognitive bias testing.

• Adult female rats had more basolateral amygdalar inflammation than males after cognitive bias testing.

• HPC neurogenesis was negatively associated to cognitive bias in young adult male rats.

**Supplementary Information:**

The online version contains supplementary material available at 10.1186/s12979-022-00299-4.

## Background

Major depressive disorder (MDD) affects 20% of the population and is characterized by an array of behavioral, emotional, and cognitive symptoms [1]. Cognitive symptoms of MDD, such as negative cognitive bias, persist in individuals in remission from MDD and are associated with increased relapse rates in these individuals [2–4]. Current treatments are not effective in reducing negative cognitive bias in MDD [5, 6] and the presence of negative cognitive bias can predict the efficacy of antidepressants in MDD [7, 8]. Thus, there is a need to develop novel therapeutics to treat MDD and attenuate negative cognitive bias in MDD. Human females are more likely to present with MDD and display cognitive symptoms of MDD compared to human males [9, 10]. Discovering the underlying mechanisms of negative cognitive bias with a focus on sex will aid in the discovery of precision treatments for negative cognitive bias in MDD.

Pattern separation, the ability to distinguish between highly similar inputs, is impaired in MDD [11–13], is involved in cognitive bias [14, 15], and relies on hippocampal neurogenesis [16–21]. Neurogenesis in the hippocampus declines with MDD and age in humans and in rodent models [22–30]. Further, treatment with antidepressants, such as selective serotonin reuptake inhibitors (SSRIs), is linked to increased neurogenesis in MDD and in rodent models with some suggestion of sex differences [23, 31, 32]. Intriguingly, there are sex differences in pattern separation and neurogenesis in response to pattern separation [33, 34]. However, the association between hippocampal neurogenesis and cognitive bias has not been examined.

Meta-analyses indicate that peripheral cytokines (including interleukin (IL)-1β, IL-6, and tumour necrosis factor (TNF)-α) and hippocampal inflammation are increased in individuals with MDD [35–38], indicating inflammation as potential biomarker for MDD. Indeed, levels of cytokines are associated with poor treatment response in individuals with MDD, indicating they may play a role in remission [39]. Moreover, there are sex and age differences in proinflammatory cytokine production with higher levels in young and middle-aged females compared to males at baseline and in response to a challenge [40–42]. However, sex differences are seldom examined in studies of inflammation in MDD, even though females may be more susceptible than males to the effects of inflammation on depressed mood [40].

Both inflammation and neurogenesis in the hippocampus influences cognition, including pattern separation [16–21, 43], indicating that both may be involved in cognitive bias. The basolateral amygdala, which is associated with mood regulation, modulates negative affect and depressive-like behavior after immune challenge [44–47] and interacts with the hippocampus to regulate neurogenesis [48]. Further, projections between the ventral hippocampus and the basolateral amygdala are required for fear memory, anxiety, and pattern separation [49–51], but sex differences have not been analyzed. Previously we found greater neural activity in dorsal and ventral hippocampal subregions (CA1, CA3, dentate gyrus) and amygdala subregions (basolateral, lateral, central) of young adult females compared to young adult males in response to a similar cognitive bias, indicating a sex difference in the role of these regions to negative cognitive bias [52]. Sex differences in the association between inflammation in the hippocampus and amygdala, neurogenesis in the hippocampus, and negative cognitive bias have yet to be examined.

In the present study, we examined sex and age differences in hippocampal neurogenesis and inflammatory cytokine (interferon gamma (IFN-γ), IL-1β, IL-4, IL-5, IL-6, IL-10, IL-13, TNF-α) and chemokine (C-X-C motif ligand 1; CXCL1) levels in the basolateral amygdala and ventral hippocampus after cognitive bias testing in rats. We hypothesized that there would be sex differences in the associations of inflammation and neurogenesis with cognitive bias. As cognitive bias changes with age, we examined adolescent, young adult, and middle-aged rats, and hypothesized that the association between cognitive bias, inflammation, and neurogenesis would differ by age.

## Results

### Males had higher inflammatory cytokines in the ventral hippocampus than females in adolescence after cognitive bias testing

**Fig. 1:**
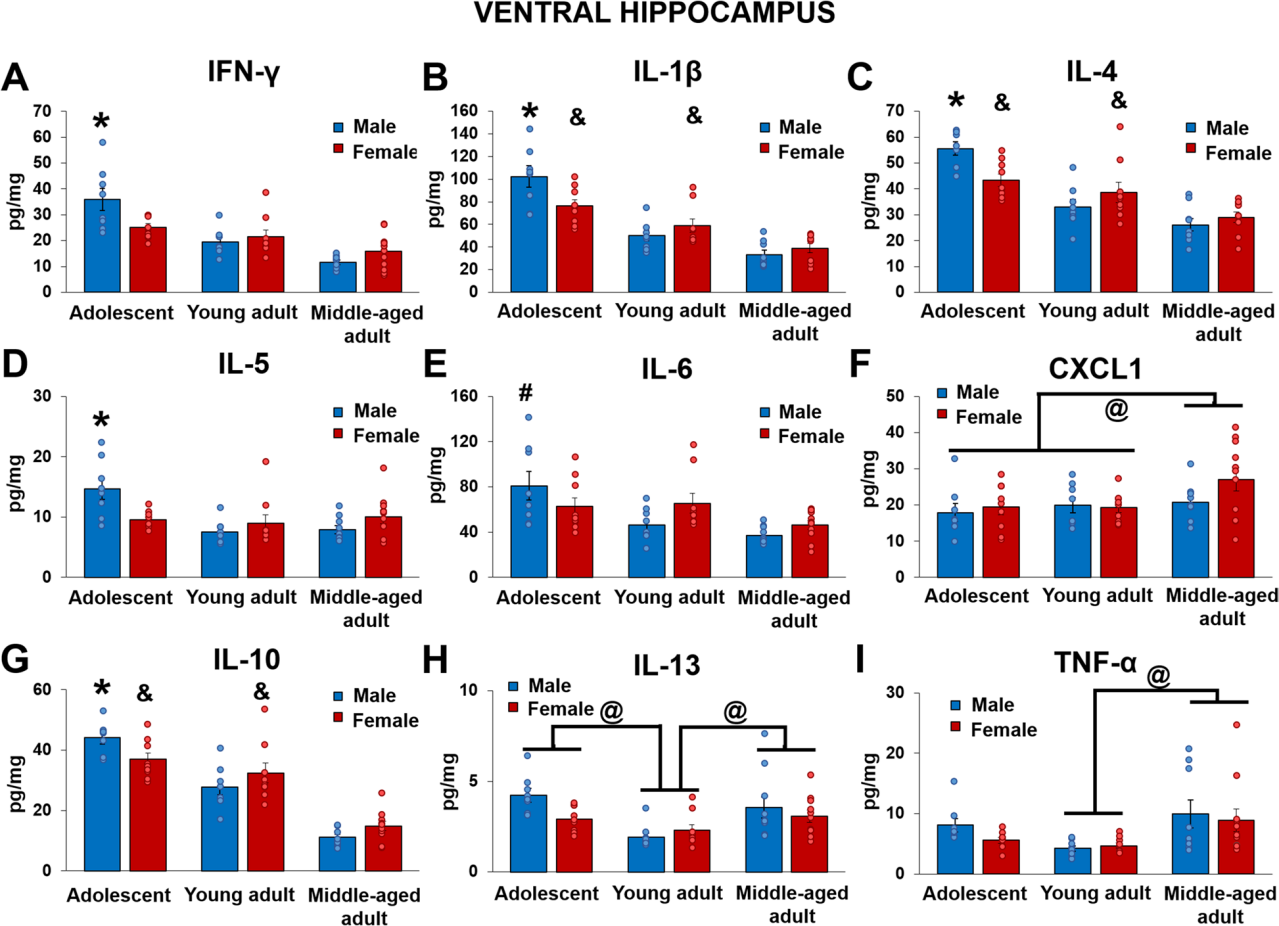
Mean (± SEM) IFN-γ (**A**), IL-1β (**B**), IL-4 (**C**), IL-5 (**D**), IL-6 (**E**), CXCL1 (**F**), IL-10 (**G**), IL-13 (**H**), TNF-α (**I**) levels in the ventral hippocampus after cognitive bias testing, normalized by total protein concentrations. Adolescent males had higher IFN- γ, IL-1β, IL-4, IL-5, and IL-10 levels compared to adolescent females and adult groups and higher IL-6 compared to young adult and middle-aged males. Adolescent and young adult females had higher IL-1β, IL-4, and IL-10 levels compared to middle-aged females. Middle-aged rats had higher CXCL1 levels compared to adolescents and young adults and higher TNF-α levels compared to young adults. Both adolescents and middle-aged adults had higher IL-13 levels than young adult rats. * indicates *p* < 0.05: compared to all other groups. & indicates *p* < 0.05: compared to middle-aged adults. # indicates *p* < 0.05: compared to young adult and middle-aged adults. @ indicates *p* < 0.05: main effects of age. *n* = 7–11. SEM = standard error of the mean

Adolescent male rats had higher IFN-γ, IL-1β, IL-4, IL-5, and IL-10 levels in the ventral hippocampus compared to adolescent females after cognitive bias testing (*p*’s < 0.006; IFN-γ: sex by age interaction: F(2,48) = 5.865, *p* = 0.005, Ƞ_p_^2^ = 0.196; IL-1β: sex by age interaction: F(2,47) = 5.557, *p* = 0.007, Ƞ_p_^2^ = 0.191; IL-4: sex by age interaction: F(2,46) = 5.683, *p* = 0.006, Ƞ_p_^2^ = 0.198; IL-5: sex by age interaction: F(2,47) = 6.229, *p* = 0.004, Ƞ_p_^2^ = 0.21; IL-10: sex by age interaction: F(2,47) = 4.352, *p* = 0.019, Ƞ_p_^2^ = 0.156). Adolescent males also had higher IFN-γ, IL-1β, IL-4, IL-5, IL-10, and IL-6 cytokine levels compared to young adult and middle-aged males (*p*’s < 0.011; IL-6: sex by age interaction: F(2,48) = 3.500, *p* = 0.038, Ƞ_p_^2^ = 0.127) and adolescent and young adult females had higher IL-1β, IL-4, and IL-10 levels compared to middle-aged females after cognitive bias testing (*p*’s < 0.046). See Fig. 1.

### Middle-aged rats had higher CXCL1, IL-13, and TNF-α levels in the ventral hippocampus compared to young adults after cognitive bias testing

Regardless of sex, middle-aged rats had higher TNF-α levels in the ventral hippocampus compared to young adults after cognitive bias testing (*p* = 0.004; main effect of age: F(2,48) = 5.96, *p* = 0.005, Ƞ_p_^2^ = 0.199). There was a trend for middle-aged rats to have higher CXCL1 levels compared to adolescents (*p* = 0.063) and young adults (*p* = 0.059) after cognitive bias testing (main effect of age: F(2,49) = 3.12, p = 0.053, Ƞ_p_^2^ = 0.113), regardless of sex. Both adolescents and middle-aged adults had higher IL-13 levels compared to young adults after cognitive bias testing (*p*’s < 0.003; main effect of age: F(2,49) = 8.06, *p* = 0.001, Ƞ_p_^2^ = 0.247). See Fig. 1.

### Females had higher inflammatory cytokines in the basolateral amygdala than males in adulthood after cognitive bias testing. Middle-aged rats had higher levels of TNF-α compared to other ages, regardless of sex

**Fig. 2:**
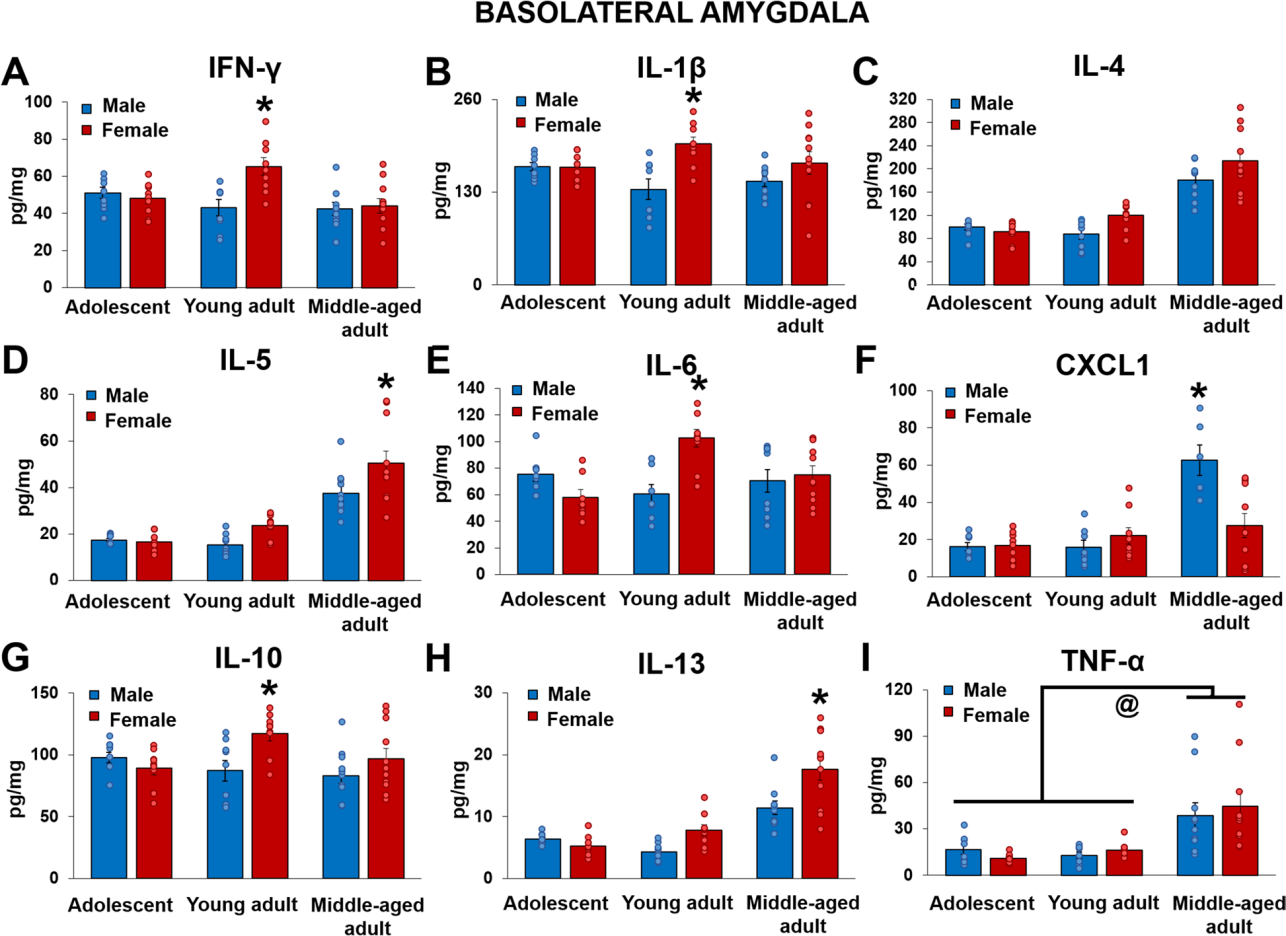
Mean (± SEM) IFN-γ (**A**), IL-1β (**B**), IL-4 (**C**), IL-5 (**D**), IL-6 (**E**), CXCL1 (**F**), IL-10 (**G**), IL-13 (**H**), TNF-α (**I**) levels in the basolateral amygdala after cognitive bias testing, normalized by total protein concentrations. Young adult females had higher IFN-γ, IL-1β, IL-6, and IL-10 levels compared young adult males and all other groups. Middle-aged females had higher levels of IL-5 and IL-13 compared to middle-aged males and all other groups, and middle-aged males had higher CXCL1 levels compared middle-aged females and to all other groups. Middle-aged adults had higher TNF-α levels compared to adolescents and young adults. * indicates *p* < 0.05: compared to all other groups. @ indicates *p* = 0.00001: main effect of age. *n* = 7–11. SEM = standard error of the mean

In contrast to the ventral hippocampus, female rats had higher levels of inflammation in the basolateral amygdala (BLA) in adulthood to middle-age than males after cognitive bias testing, depending on the cytokine. Young adult female rats had higher levels of IFN-γ, IL-1β, IL-6, and IL-10 than young adult males [IFN-γ (*p* = 0.002; sex by age interaction: F(2,49) = 5.8003, *p* = 0.006, Ƞ_p_^2^ = 0.191), IL-1β (*p* = 0.005; sex by age interaction: F(2,47) = 3.622, *p* = 0.034, Ƞ_p_^2^ = 0.134), IL-6 (*p* = 0.001; sex by age interaction: F(2,46) = 8.44, *p* = 0.0008, Ƞ_p_^2^ = 0.268), and IL-10 (*p* = 0.024; sex by age interaction: F(2,49) = 3.828, *p* = 0.029, Ƞ_p_^2^ = 0.135), IL-5 (*p* = 0.0003; sex by age interaction approached significance: F(2,49) = 2.618, *p* = 0.08, Ƞ_p_^2^ = 0.097), IL-13 (*p* = 0.002; sex by age interaction: F(2,49) = 5.482, *p* = 0.007, Ƞ_p_^2^ = 0.183)]. There were no sex differences in these cytokines in adolescent or middle-aged rats (*p*’s > 0.424). Furthermore, young adult females had higher IFN-γ, IL-6, and IL-10 levels compared to all other groups (*p*’s < 0.048, although *p* = 0.09 compared to middle-aged females for IL-10). Middle-aged females had higher levels of IL-5 and IL-13 than middle-aged males and all other groups (*p*’s < 0.0002). Middle-aged males had higher CXCL1 levels in the BLA compared to middle-aged females and all other groups (*p*’s < 0.0002; sex by age interaction: F(2,44) = 10.03, *p* = 0.0003, Ƞ_p_^2^ = 0.313). Regardless of sex, middle-aged rats had higher TNF-α levels in the BLA compared to adolescents and young adults (*p*’s < 0.0002; main effect of age: F(2,48) = 14.81, *p* = 0.00001, Ƞ_p_^2^ = 0.382). See Fig. 2.

### Negative correlations between basolateral amygdala and ventral hippocampal cytokines in young adulthood, sex difference in correlations between basolateral amygdala IL-6 and ventral hippocampus cytokines in adolescence after cognitive bias testing

Correlations of cytokine and CXCL1 levels within the BLA and the ventral hippocampus were largely positive in all age groups after cognitive bias testing, although correlations between regions were more negative in young adult rats, regardless of sex, compared to the other age groups. In adolescence, there was a sex difference in the correlations between BLA IL-6 and cytokines in the ventral hippocampus, with positive correlations in adolescent female rats compared to negative correlations in adolescent male rats (sex difference in BLA IL-6 and ventral hippocampal IFN-γ (z = 2.768, *p* = 0.003), IL-1β (z = 3.335, *p* < 0.001), IL-4 (z = -3.464, *p* < 0.001), IL-5 (z = 1.979, *p* = 0.024), IL-10 (z = -2.694, *p* = 0.004), IL-13 (z = 2.767, *p* = 0.003), TNF-α (z = -2.727, *p* = 0.003)). See supplementary Fig. S1.

We next correlated negative cognitive bias score with inflammatory markers in both the BLA or hippocampus but no correlations survived Bonferroni correction. See supplemental Table S1.

### Neurogenesis decreased with age and increased with cognitive bias testing in younger animals

As expected, adolescent rats had higher DCX expression compared to the adult groups, regardless of sex and region (*p*’s < 0.00013). Adolescent and young adult rats also had higher DCX expression in the dorsal hippocampus compared to the ventral hippocampus (*p*’s < 0.004) regardless of sex, but this was not found in middle-aged rats (*p* = 0.403; region by age interaction: F(2,73) = 12.47, *p* = 0.00003, Ƞ_p_^2^ = 0.255).

**Fig. 3:**
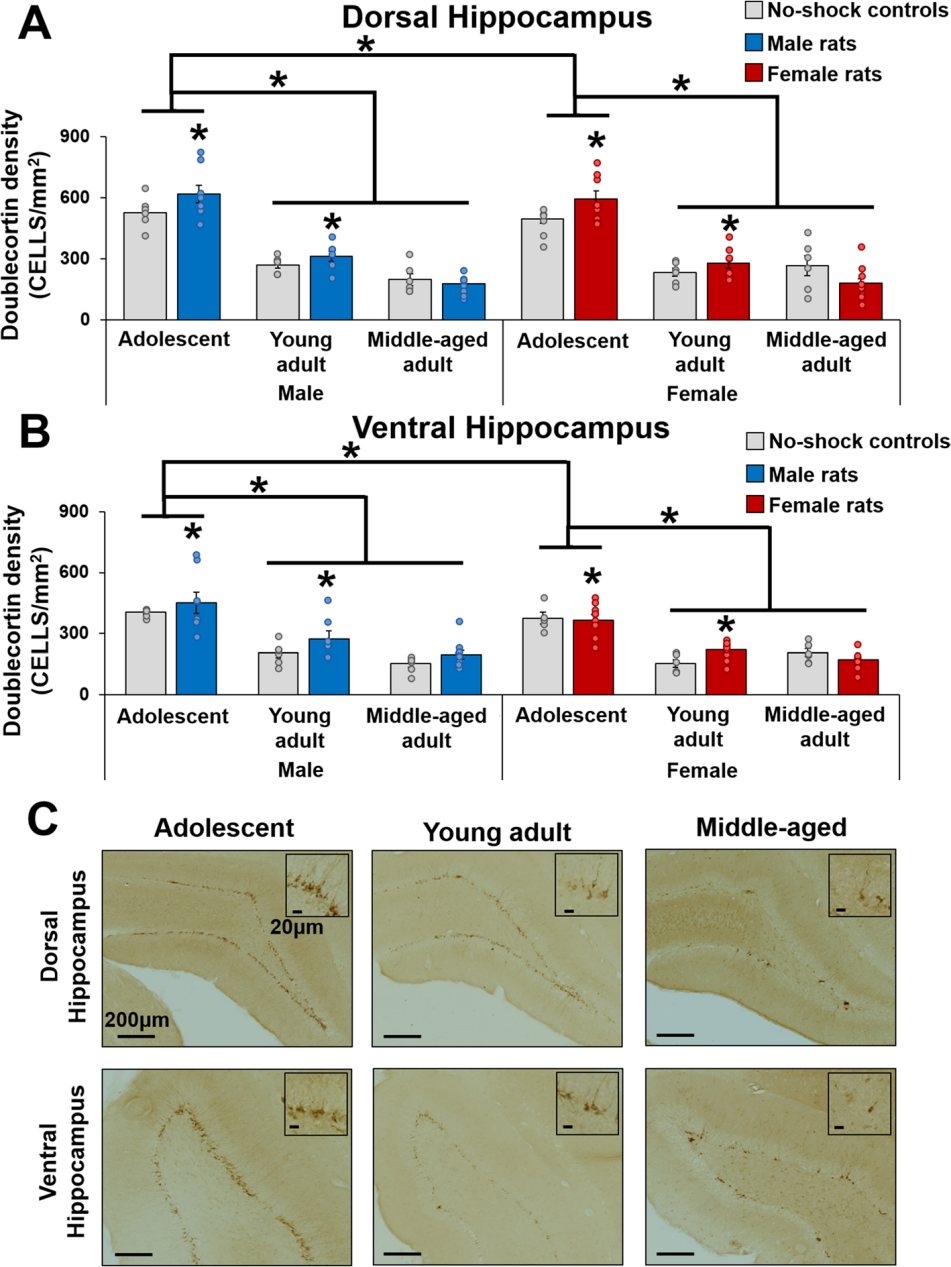
Mean (± SEM) doublecortin (DCX) expression in the dorsal hippocampus (**A**) and ventral hippocampus (**B**) of male and female adolescent, young adult, and middle-aged rats that underwent the cognitive bias procedure with or without footshocks (no-shock controls). Representative images of DCX expressing cells in the granule cell layer of the dorsal and ventral hippocampus of adolescent, young adult, and middle-aged rats (**C**). DCX expression is higher in adolescents compared to adults regardless of sex, and adolescent males have higher DCX expression compared to adolescent females regardless of region. Furthermore, adolescents and young adults have higher DCX expression after cognitive bias testing compared to no-shock controls regardless of region. Adolescent and young adult rats also have higher DCX expression in the dorsal hippocampus compared to the ventral hippocampus. *indicates *p*’s < 0.02. *n* = 5–6 for no-shock controls, *n* = 7–11 for test rats. SEM = standard error of the mean

Rats that underwent cognitive bias testing had higher DCX expression than no-shock controls in adolescence (*p* = 0.015) and young adulthood (*p* = 0.019) regardless of sex and region, but not in middle-age (*p* = 0.274) (condition by age interaction: F(2,73) = 4.439, *p* = 0.015, Ƞ_p_^2^ = 0.108). Moreover, adolescent males had higher DCX expression than adolescent females (*p* = 0.042) regardless of region, not found in adult groups (*p*’s > 0.062) (sex by age interaction: F(2,73) = 3.273, *p* = 0.044, Ƞ_p_^2^ = 0.082). See Fig. 3.

### Negative cognitive bias was negatively associated with dorsal neurogenesis in young adult males

Dorsal DCX expression was negatively correlated with freezing (*r* = -0.787, *p* = 0.036) and negative cognitive bias score (*r* = -0.7643, *p* = 0.045) in young adult male rats only as there were no significant correlations with freezing or negative cognitive bias in any other group (*p*’s > 0.241). However, these correlations do not survive Bonferroni correction (See supplemental Table S2).

### IL-13 levels in the ventral hippocampus were associated ventral hippocampal neurogenesis in males

In male rats, ventral hippocampal DCX expression was positively associated with ventral hippocampal IL-13 and TNF-α, but dorsal hippocampal DCX expression was negatively associated with BLA IL-1β (*p*’s < 0.043). However, only the correlation between ventral hippocampal DCX expression and IL-13 survived Bonferroni (*p* = 0.001). In female rats, dorsal hippocampal DCX expression was positively associated with ventral hippocampal IFN-γ, IL-5, IL-13, and CXCL1 in young adulthood (*p*’s < 0.03). These positive correlations were also seen in female middle-aged rats in the BLA, with dorsal hippocampal DCX expression and BLA IL-4, IL-5, IL-10, and IL-13 (*p*’s < 0.043). But none of these correlations in females survived the Bonferroni correction (see supplemental Table S3).

### Associations between cytokine/chemokine levels differ by age and sex after cognitive bias testing

**Fig. 4:**
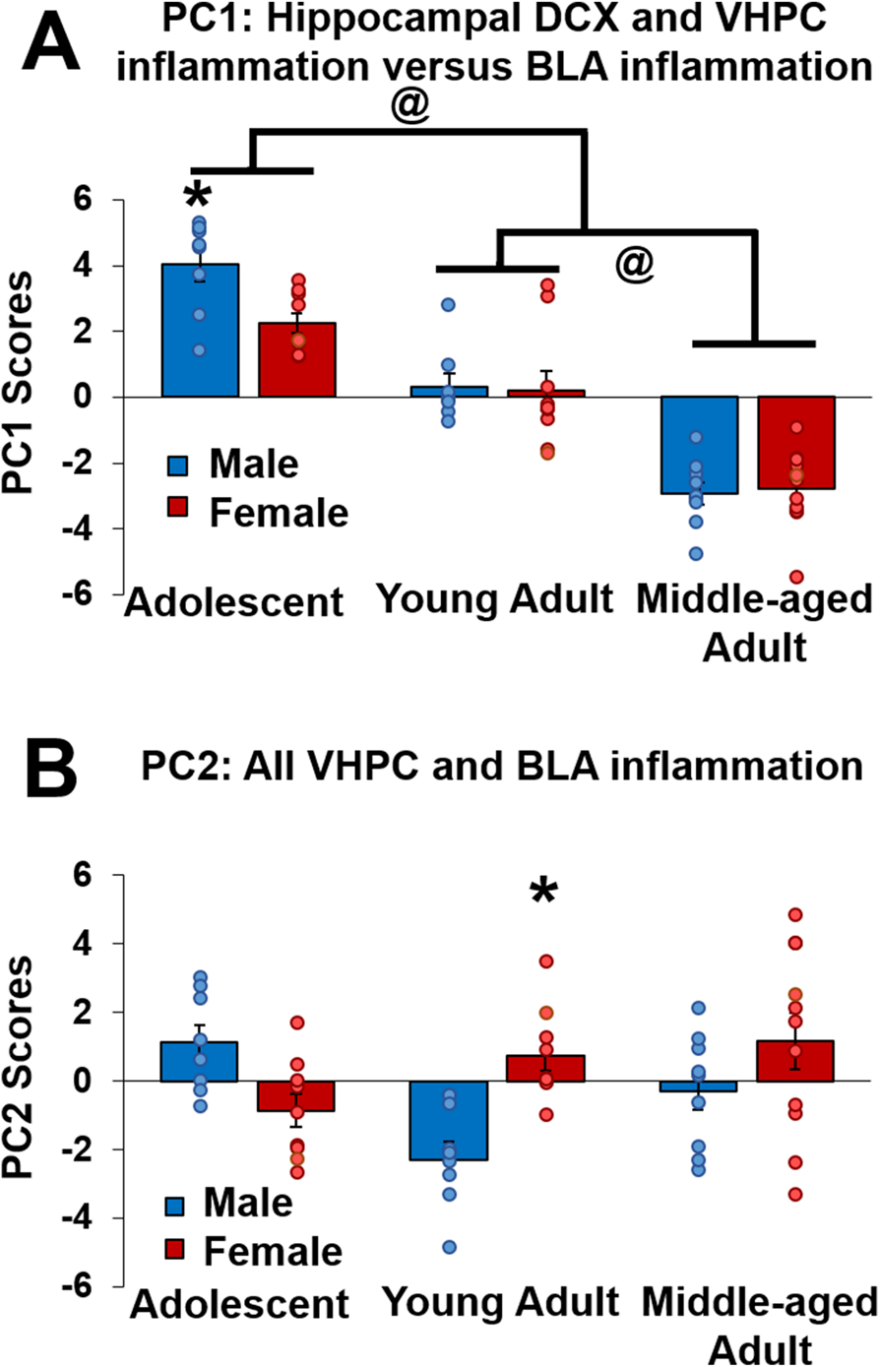
Mean (± SEM) principal component (PC) scores for doublecortin (DCX) in the dorsal and ventral hippocampus, ventral hippocampus (VHPC) cytokine/chemokine levels, and basolateral amygdala (BLA) cytokine/chemokine levels. PC1 scores (**A**), associated with hippocampal DCX and VHPC cytokines compared to BLA cytokines, found higher hippocampal DCX and VHPC cytokine/chemokine levels in adolescent males compared to adolescent females and in adolescents compared to adult groups. Further BLA cytokines/chemokines were higher in middle-aged rats compared to young rats. @indicates *p* < 0.00014: comparison between each age group, *indicates *p* < 0.006: compared to adolescent females. PC2 scores (**B**), associated with VHPC and BLA cytokine/chemokine levels, were more involved in young adult females compared to young adult males. *indicates *p* < 0.005: compared to young adult males. *n* = 8–11 per sex/age. SEM = standard error of the mean

Principal component analysis was used to identify clusters/pathways of interest or components [53–55]. The first two principal components accounted for 60.43% of the variance of all cytokine/chemokine and neurogenesis data. Component 1 accounted for 38.66% of the variance and was associated with cytokine/chemokine levels in the ventral HPC and hippocampal neurogenesis compared to cytokine/chemokine levels in the BLA. Component 2 accounted for 21.78% of the variance and was associated with all cytokines/chemokine levels in both regions. The loadings for PC1 and PC2 are shown in Table 1. An ANOVA on Principal Component 1 found that hippocampal inflammation and neurogenesis were higher in adolescents compared to the adult age groups (*p*’s < 0.00013), and IL-4, L-5, IL-13, TNF-α, and CXCL1 levels in the BLA were higher in middle-aged rats compared to the younger age groups (*p*’s < 0.00013) after cognitive bias testing (main effect of age: F(2,48) = 100.5, *p* < 0.000001, Ƞ_p_^2^ = 0.807). A priori we expected sex differences and hippocampal inflammation and neurogenesis were elevated in adolescent males compared to adolescent females (*p* < 0.006; sex by age interaction: F(2,48) = 2.851, *p* < 0.068, Ƞ_p_^2^ = 0.106) with no other sex differences seen. There was no significant main effect of sex (*p* > 0.102; see Fig. 4A). An ANOVA on Principal Component 2 found higher cytokine associations in young adult females compared to males (*p* = 0.005) after cognitive bias testing (sex by age interaction: F(2,48) = 8.578, *p* = 0.0007, Ƞ_p_^2^ = 0.263). There were no other significant main or interaction effects (all *p*’s > 0.09). See Fig. 4B.

**Table 1:** Principal component loadings from the doublecortin (DCX) and cytokine/chemokine data

		PC1	PC2
Dorsal hippocampus	DCX	0.792^*^	-0.034
Ventral hippocampus	DCX	0.735^*^	-0.033
	IFN-γ	0.846^*^	0.383^*^
	IL-1β	0.910^*^	0.317^*^
	IL-4	0.904^*^	0.312^*^
	IL-5	0.593^*^	0.584^*^
	IL-6	0.703^*^	0.338^*^
	CXCL1	-0.173	0.381^*^
	IL-10	0.937^*^	0.189
	IL-13	0.424^*^	0.591^*^
	TNF-α	-0.092	0.484^*^
Basolateral amygdala	IFN-γ	0.04	0.592^*^
	IL-1β	-0.064	0.716^*^
	IL-4	-0.761^*^	0.537^*^
	IL-5	-0.743^*^	0.543^*^
	IL-6	-0.201	0.567^*^
	CXCL1	-0.567^*^	0.171
	IL-10	-0.099	0.681^*^
	IL-13	-0.678^*^	0.585^*^
	TNF-α	-0.542^*^	0.433^*^

Significant loadings are indicated in bold

^*^indicates significance at *p* < 0.022

**Table 2:**
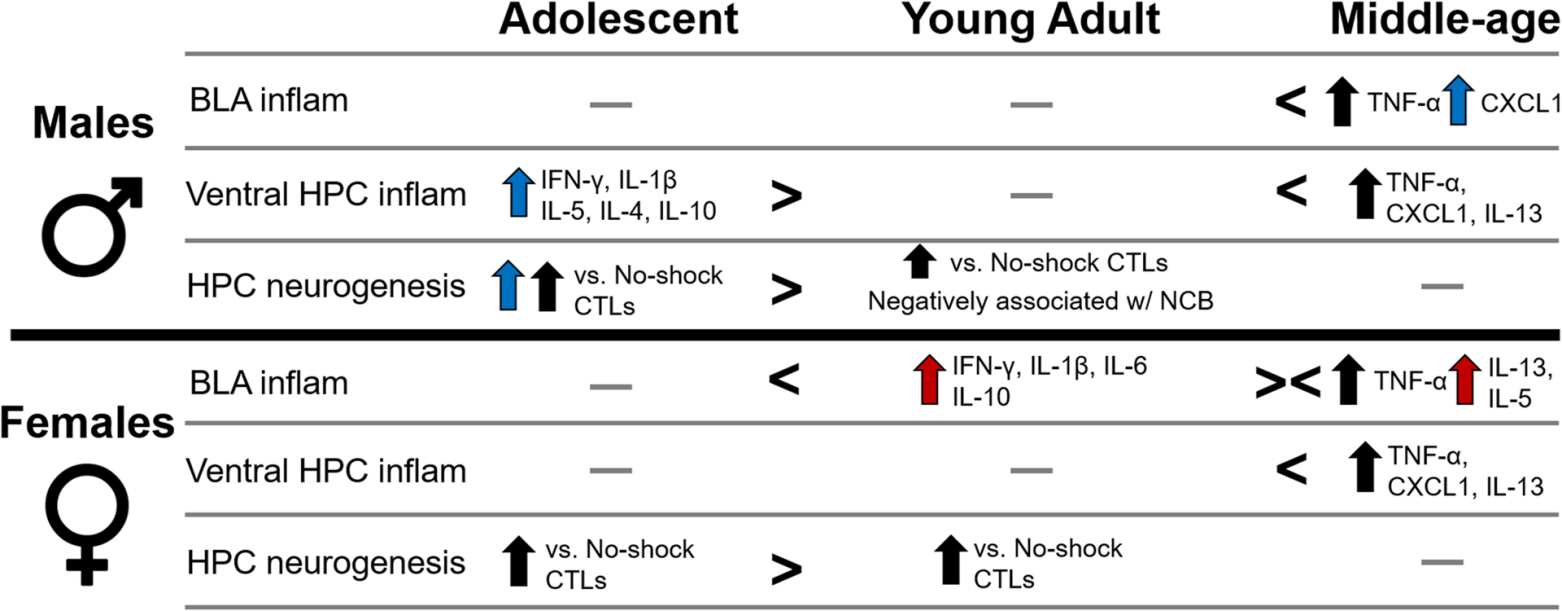
Summary of age and sex specific inflammation and neurogenesis results in rats after cognitive bias testing

Blue arrows indicate higher in males than females. Red arrows indicate higher in females than in males. Black arrows indicate higher regardless of sex. > or < indicate higher or lower than adjacent age groups. *Inflam* inflammation, *NCB* negative cognitive bias, *BLA* basolateral amygdala, *HPC* hippocampus

## Discussion

Here, we report sex and age differences in cytokine and chemokine levels after cognitive bias testing that are dependent on region. Adolescent male rats had higher levels of cytokines in the ventral hippocampus than females, but adult (young and middle-age) females had higher levels of cytokines than adult males in the BLA. Furthermore, middle-aged rats had higher levels of TNF-α and the chemokine CXCL1 in both the hippocampus and amygdala after cognitive bias testing than all other ages. Middle-aged rats also had higher levels of IL-13 compared to younger rats in the ventral hippocampus after cognitive bias testing. Negative correlations between basolateral amygdala and ventral hippocampal cytokines were found in young adults after cognitive bias testing. Whereas adolescent males had negative correlations, adolescent females had positive correlations between IL-6 levels in the basolateral amygdala and ventral hippocampal cytokines after cognitive bias testing. Principal component analyses found high ventral hippocampus cytokine/chemokine levels in adolescents, high basolateral amygdala cytokine/chemokine levels in middle-aged rats, and that young adult females had higher levels of inflammation than young adult males after cognitive bias testing. When examining hippocampal neurogenesis, we found higher DCX expression in adolescent males compared to females and higher DCX expression after cognitive bias testing in adolescents and young adults compared to no-shock controls. These findings demonstrate sex and age differences in possible biomarkers (inflammation, neurogenesis) related to negative cognitive bias and regional differences in these results (BLA in females, HPC in males). Future studies should examine these biomarkers under stress conditions to determine their roles in rats displaying a depressive-like endophenotype. See Table 2 for a summary.

### Adolescent males had high ventral hippocampal inflammation after cognitive bias testing

Adolescent male rats have higher IFN-γ, IL-1β, IL-4, IL-5, and IL-10 levels than adolescent females in the ventral hippocampus after cognitive bias testing. Both increased inflammation (IL-1β, IL-1α) and neural activation in the ventral hippocampus are linked to an increased susceptibility to a stressor, with increased depressive-like behaviors in male rats [56–58]. In the current study, negative cognitive bias was positively associated with IL-4 and IL-10 in the ventral hippocampus of adolescent males only, suggesting a greater role of hippocampal inflammation for this particular depressive-like endophenotype in adolescent males compared to all other groups. Overall, adolescent rats had higher cytokine levels in the ventral hippocampus than the adult groups. This is similar to past findings in naïve male rats as there were decreased IL-4 levels with age in both plasma [59] and hippocampus (4 months to 22–23 months; [60, 61]), suggesting the decrease in IL-4 in the present study was not due to testing alone. Put together, these data suggest a greater role of cytokine levels in the hippocampus of adolescents for certain behaviors and specifically in adolescent males compared to females. Future studies examining the role of inflammation in depressive-like behavior should take age and sex into account.

### Young adult females had higher basolateral amygdala inflammation than young adult males

Young adult female rats had higher IFN-γ, IL-1β, IL-6, and IL-10 in the basolateral amygdala compared to young adult males. Higher plasma IL-6 is seen in adult women compared to men at baseline [42] and there is an up-regulation of genes related to inflammation in the brain in women compared to men [62]. In naive mice, higher IL-6 was reported in the ventral hippocampus of aged female mice compared to aged male mice [63]. However, few papers have examined sex differences in the role of inflammation in depressive-like behavior or the role of inflammation in the basolateral amygdala in cognition. Our data suggests a greater role of cytokines in the basolateral amygdala of females than in males in cognitive bias.

### Neurogenesis was related to negative cognitive bias in males but not in females

In the present study, we found that rats that underwent cognitive bias testing had higher levels of hippocampal neurogenesis than no-shock control rats dependent on age and regardless of sex, which could be a result of cognitive training to boost neurogenesis [33, 64]. We also found higher DCX expression in adolescent males compared to adolescent females, similar to findings of greater hippocampal neurogenesis in naïve pre-pubertal males compared to females [65, 66]. Intriguingly, reduced hippocampal neurogenesis was associated with increased negative cognitive bias in young adult males only which may be consistent with findings of increased hippocampal neurogenesis being associated reduced depressive-like behavior, found after antidepressant treatment or voluntary exercise in both males and females [24, 27, 31, 32, 67]. It is difficult to understand why we saw this association in males but not in females in the present study. Spatial, but not contextual, pattern separation training increases neurogenesis in male but not female rats [33, 34]. The paradigm used in the present study relies on contextual pattern separation and we found that cognitive training (with shock) was sufficient to boost neurogenesis in both males and females but was related to negative cognitive bias only in males. Chronic stress reduces neurogenesis in the hippocampus of both males and females [68–71], however we do not think the shocks in this study acted as a stressor as we saw enhanced neurogenesis (and no other somatic indices of depressive-like endophenotypes) in adolescent and young adult rats with cognitive bias training compared to no-shock controls. Future studies should examine possible sex differences after chronic stress on negative cognitive bias and the role of neurogenesis in the hippocampus.

### Age influences cytokines and neurogenesis

Aging was associated with a decline in neurogenesis in the hippocampus, which was expected given that a decline in hippocampal neurogenesis is noted across the lifespan in humans, rodents, and non-human primates [72–76]. Depending on the age, sex, and inflammatory signal we saw decreases in anti-inflammatory IL-4, IL-5, and Il-10 levels from adolescence to adulthood particularly in vHPC which partially mirrors the literature. For example, hippocampal IL-4 and IL-5 decreases with age in naïve male rodents [59–61, 77]. But other inflammatory markers showed an increase with age in the hippocampus and basolateral amygdala. Middle-aged rats had higher TNF-α and CXCL1 in the ventral hippocampus and basolateral amygdala than younger groups after cognitive bias testing. These findings are similar to past findings of increased immune-related genes with age in the hippocampus and cortex of humans and mice [62, 78, 79] that had not undergone cognitive bias testing. Along with TNF-α, the chemokine CXCL1 was also increased with age, particularly in the BLA of males in the present study. Both TNF-α and CXCL1 in the cerebrospinal fluid are upregulated after chronic stress in adult male mice [80]. These data suggest a greater role of hippocampal TNF-α and CXCL1 with age and stress. In addition, increased CXCL1 in the BLA of middle-aged males compared to middle-aged females may play a role in greater negative cognitive bias in middle-aged males as we found in a previous study that negative bias was increased in males relative to females in middle age only [52] and warrants further investigation. Overall, these data stress the importance of examining age and sex when exploring the link between inflammation and depressive-like cognitive endophenotypes.

### Implications

Meta-analyses find that plasma immune signalling (increased IL-6, IL-10, or TNF-α) are associated with increased negative mood and negative cognitive bias in humans [81], but so far studies have yet to examine the association between negative cognitive bias and inflammation in the brain. In our study, inflammation in the brain did not significantly correlate with negative cognitive bias, although there were positive correlations between IL-10 and IL-4 in the BLA of adolescent males that did not survive Bonferroni correction. Fluoxetine injections or CXC receptor 2 inhibitor injections reduce CXCL1 and depressive-like behavior in adult male mice [80], and our data suggest that these treatments might influence negative cognitive bias in middle-aged groups. Because negative cognitive bias is a key factor for an increased risk for MDD, symptom severity, and relapse [82–86] it is crucial to discover novel therapeutic targets for this cognitive symptom of MDD. Our data stresses the fact that sex and age need to be considered when investigating novel therapeutic targets for negative cognitive bias and related mechanisms.

This cognitive bias procedure was chosen because it measures the degree of negative cognitive bias after evaluating an ambiguous situation and the rats learn this procedure quickly compared to other cognitive bias procedures (within 16 days versus several weeks; [87]). Similar brain regions involved in human cognitive bias are involved in the display of this cognitive bias after this task in rats [52]. As discussed above, rats that undergo this cognitive bias procedure have similar sex and age differences in basal inflammation that have been found in naïve rodents and non-stressed humans [42, 59, 60, 60, 62, 77].

Although stress can influence inflammation in an age and sex-dependent manner in rodents and humans [88–94] we do not believe that the use of footshocks in this cognitive bias procedure is overly stressful. Footshocks in the current study are less intense (3 × predictable 0.6 mA footshocks, 2 s in duration, per session) than footshock paradigms that increase inflammation (5 × unpredictable 0.8 mA footshocks, 8 s in duration, per session; [70]) or depressive-like behaviors (5–60 × unpredictable 0.8–1.5 mA, 8–10 s duration, per session; [95–98]) in rats. In addition, rats that underwent the cognitive bias procedure in the current study had higher or no change in hippocampal neurogenesis compared to no-shock controls depending on age, suggesting that this procedure might be more enriching than stressful. Furthermore, not all rats that undergo this procedure display a negative cognitive bias and both body mass and relative adrenal mass did not differ between no-shock controls and rats that undergo cognitive bias testing with footshocks [52]. Put together, these data suggest that this cognitive bias procedure itself is likely not overly stressful.

## Conclusion

Overall, inflammation in the brain reverses from higher in males in the ventral hippocampus during adolescence to higher in females in the basolateral amygdala during adulthood after cognitive bias testing compared to the opposite sex. Moreover, hippocampal neurogenesis is higher in adolescent males than adolescent females and hippocampal neurogenesis is associated with reduced negative cognitive bias in young adult males. Future studies should examine whether models of depression, such as chronic unpredictable stress, increase negative cognitive bias and whether antidepressants or exercise will reverse effects on negative bias in an age by sex manner. These data provide potential biomarker targets to reduce negative cognitive bias in MDD that vary by age and sex.

## Methods

### Animals

Male and female Sprague–Dawley rats (*N* = 91) were bred in house from animals obtained from Charles River (Québec, Canada). Only 1 male and 1 female rat per litter was assigned to each age group and each condition to avoid litter confounding effects. Males and females were housed (2–3 per cage) in separate colony rooms. Rats were maintained under a 12 h light–dark cycle, with lights on at 07:00 h. Rats were housed in opaque polyurethane bins (48 × 27 × 20 cm) with aspen chip bedding and ad libitum access to autoclaved tap water and rat chow (Jamieson’s Pet Food Distributors Ltd, Delta, BC, Canada). Rats were left undisturbed, apart from weekly cage changing, until they reached the correct age for testing. All experimental procedures were approved by the University of British Columbia Animal Care Committee and in accordance with the Canadian Council on Animal Care guidelines.

### Cognitive bias task procedure

Cognitive bias procedure and tissue collection methods are previously described in Hodges et al. ([52]). Briefly, male and female rats were randomly assigned to be tested in adolescence (postnatal day (PD) 40, *n* = 29), young adulthood (PD 100, *n* = 30), or middle-aged adulthood (PD 210, *n* = 36) and then to one of the two groups—test rats (adolescents: male *n* = 8, female *n* = 9; young adults: *n* = 9 per sex; middle-aged adults: *n* = 12 per sex), or no-shock controls (*n* = 6 per sex and age). Rats were placed in a shock-paired context (Context A) and in a no-shock-paired context (Context B) for 5 min each daily for 16 consecutive days, one context in the morning (8:30 h – 11:00 h) and the other context in the afternoon (13:00 h – 15:30 h). After 16 days of training, rats were placed in an ambiguous context (Context C) for 5 min with no footshock on Test Day (Day 18). Context C partially resembled both Contexts A and B in terms of transport (duration and method), illumination (two lights), one lever out, and an intermediate pattern of lines on the walls (7 mm between lines). No-shock controls did not receive a footshock in any context. Time spent freezing (no head or body movement besides breathing; [99]) during the first 3 min of entering each context was measured on each day and percentage freezing was computed. Further, a difference score was created by subtracting percentage freezing in Context C on Day 18 from percentage freezing in Context B (no footshock-paired) on Day 16 and used to index negative cognitive bias scores (high freezing = negative cognitive bias; low freezing = neutral/positive cognitive bias; adapted from [100, 101]).

These behavioral data were published previously [52]. We found that adolescent rats had a more positive cognitive bias compared to a greater negative cognitive bias in adults and middle-aged males had a greater negative cognitive bias than middle-aged females (see supplementary Fig. S2). Regardless of age and sex, test rats had higher freezing than no-shock controls in the ambiguous context. Ninety min after exposure to Context C on day 18, test rats were euthanized by decapitation. Brains were removed from the skull and cut in equal halves along the sagittal plane. The left hemisphere was used for DCX immunohistochemistry and the right hemisphere was used for electrochemiluminescence (described below).

### DCX Immunohistochemistry

We examined hippocampal neurogenesis using a marker of immature neurons and microtubule-associated protein, doublecortin (DCX; [102]) in both the cognitive bias and no-shock controls. The left hemisphere was placed into a 4% paraformaldehyde solution for 24 h, and subsequently placed into a 30% sucrose in 0.1 M phosphate buffered saline (PBS; pH 7.4) for another 24 h and then until sliced. Coronal Sects. (30 µm) were sliced on a microtome and collected from approximately bregma 3.72 mm to -6.96 mm [103]. Sections were stored in an antifreeze solution (30% ethylene glycol, 20% glycerol in 0.1 M phosphate buffer (PB; pH 7.4)) at -20 °C until immunohistochemistry assays were conducted.

Coronal sections were successively washed 3 × in PBS for 10 min per wash and incubated at room temperature in a 0.6% hydrogen peroxide (H2O2; H1009, Sigma-Alrich, St. Louis, MO, USA) in distilled water (dH2O) for 30 min. Sections were then washed another 3 × in 0.1 M PBS for 10 min per wash, and then incubated at 4 °C in DCX primary antibody (1:1000 goat Anti-DCX pAb; SC-8066; Santa Cruz Biotechnology, Dallas, TX, USA), 3% normal rabbit serum (VECTS5000, Vector Laboratories, Inc, Burlingame, CA), and 4% Triton-X in PBS for 24 h. The next day, sections were washed 5 × in 0.1 M PBS for 10 min per wash and incubated overnight at 4 °C in secondary antibody (biotinylated rabbit anti-goat IgG; 1:500; Vector Laboratories, Inc, Burlingame, CA). The last day, after another series of 5 washes in 0.1 M PBS for 10 min per wash, sections were incubated in an avidin–biotin horseradish peroxidase solution (PK-4000, Vector Laboratories, Inc, Burlingame, CA) for 4 h at room temperature. Sections were washed 3 × in 0.1 M PBS for 10 min per wash and horseradish peroxidase was visualized using 3,3’ diaminobenzidine (DAB) in a 3 M sodium acetate buffer containing 2.5% nickel sulfate and 0.05% H2O2 (SK-4100, Vector Laboratories, Inc, Burlingame, CA) for 3 min. Sections were washed another 3 × in 0.1 M PBS for 10 min per wash and then mounted on Superfrost Plus slides (Fisher Scientific, Inc., Hampton, NH) and let dry. Sections were then dehydrated using increasing concentrations of ethanol (50%, 70%, 95%, 100% for 2, 2, 2, and 10 min respectively), and then cleared with xylene for 10 min and coverslipped using Permount mounting medium (Fisher Scientific, Inc., Hampton, NH).

DCX protein immunostained brain sections were analyzed using a Nikon Eclipse 80i microscope in the dorsal hippocampus (within bregma -2.64 mm and -4.56 mm) and ventral hippocampus (within bregma -5.76 mm and -6.36 mm). Photomicrographs were taken using a slidescanner (ZEISS Axioscan 7 Slide Scanner, Germany) and used to trace outline of each subregion of interest to calculate the area of each region using ImageJ software (Image J, 2020). Cell counts of DCX expressing cells were conducted by experimenters’ blind to experimental condition and averaged across 2 sections per animal hippocampal region using a 40 × objective. DCX expressing cells for each subregion of interest was calculated by dividing the cell count by the corresponding area in mm^2^ for each animal.

### Multiplex cytokine electrochemiluminescence

Electrochemiluminescence was done in accordance with previous protocols [104]. The right hemisphere of the brain was rapidly frozen and coronally sliced at 300 µm. The BLA (within bregma 1.92 mm and 0.96 mm) and the vHPC (within -5.76 mm and -6.36 mm) were identified and dissected out using tissue punching tools (0.75 mm, 1.20 mm, and 2 mm in diameter; Harris Uni-Core, Sigma-Alrich) and placed directly into tubes containing beads (1.4 mm ceramic spheres, Lysing Matrix D, MP Biomedicals™, Santa Ana, CA, USA) on dry ice. Tissue was homogenized in complete lysis buffer using the Omni Bead Ruptor 24 (Omni International. Kennesaw, GA, USA). After homogenization, samples were centrifuged at 4°C at 1000 g for 10 min and supernatant was collected and stored at -80°C until cytokine analysis.

Cytokine levels were quantified in samples using a multiplex electrochemiluminescence immunoassay kit (V-PLEX Proinflammatory Panel 2, Rat) from Meso Scale Discovery (Rockville, MD, USA). The following 8 cytokines and 1 chemokine were quantified in each sample: interferon gamma (IFN- γ), interleukin (IL)-1β, IL-4, IL-5, IL-6, IL-10, IL-13, tumor necrosis factor (TNF)-α, and the chemokine C-X-C motif ligand 1 (CXCL1). Samples were run in duplicates and plates were read using a Sector Imager 2400 (Meso Scale Discovery) and analyzed using the Discovery Workbench 4.0 software (Meso Scale Discovery). The lower limits of detection (LLOD) were as follows for each individual plate (4 plates total) in pg/mL: IFN- γ: 0.674, 1.776, 1.62, 2.652; IL-1β: 1.995, 3.745, 3.616, 8.118; IL-4: 1.64, 4.613, 2.062, 5.75; IL-5: 0.552, 1.563, 0.541, 0.999; IL-6: 2.18, 4.09, 2.462, 3.718; IL-10: 0.789, 1.99, 1.744; 5.574; IL-13: 0.168, 0.698, 0.143, 0.252; TNF-α: 0.385, 0.97, 0.298, 0.399; and CXCL1: 0.99, 0.406, 0.967, 0.558. Inter-assay coefficient of variation was < 23% for all cytokines between plates.

### Data analyses

General linear mixed model ANOVAs for levels of each cytokine/chemokine in the basolateral amygdala and ventral hippocampus were run with sex (male, female) and age (adolescence, young adulthood, middle-aged adulthood) as between-subjects factors. A repeated measures ANOVA using the same between-subjects factors as above and condition (no-shock controls, test rats) as an additional between-subjects factor was performed on the dorsal and ventral hippocampus DCX data. Pearson’s correlations were conducted between BLA or vHPC cytokine/chemokine levels, dorsal or ventral hippocampal DCX, and freezing in the ambiguous context or negative cognitive bias score. Principal component analyses were performed using DCX data and inflammation data in each brain region in test rats only. Missing values, due to outliers (two standard deviations below or above the mean), which accounted for 1.65% of the data, were replaced by the mean for PCA analyses. One middle-aged male was completely removed from PCA analyses because they were missing 78% of cytokine/chemokine data in the ventral hippocampus due to cytokine levels two standard deviations above the mean. Post-hoc tests used Newman-Keuls comparisons. Any a priori comparisons examining sex differences were subjected to Bonferroni comparisons. Significance level of *p* < 0.05 was used. All statistical analyses were performed using Statistica software (v. 9, StatSoft, Inc., Tulsa, OK, USA).

Four test rats were excluded from the following analyses due to their inability to distinguish between the shock- and no-shock-paired contexts on Day 16 of training (2 middle-aged males, 1 middle-aged female, 1 young adult male).

## Supplementary Information

**Additional file 1:** **Fig. S1.** Heatmaps showing all correlations and cytoscape graphs showing significant correlations ≥0.7 between inflammatory marker correlations between the ventral hippocampus and basolateral amygdala in male and female adolescent (A-C), young adult (D-F), and middle-aged (G-I) rats after cognitive bias testing. The thickness of the lines in B, C, E, F, H, and I are related to the strength of the correlation (stronger is thicker), whereas the color relates to the valence (positive (red) or negative (blue)) of the correlation. Correlations of inflammatory marker levels within the ventral hippocampus and basolateral amygdala were largely positive in all age groups, although correlations between regions were more negative in male and female young adults compared to the other age groups. In adolescence, there was a sex difference in the correlations between basolateral amygdala IL-6 and cytokines (IFN-γ, IL-1β, IL-4, IL-5, IL-10, IL-13, TNF-α) in the ventral hippocampus, with positive correlations in adolescent females compared to negative correlations in adolescent males. *n*=7-11 per group. **Fig. S2.** Mean (±SEM) percentage of time spent freezing (A) and negative cognitive bias discrimination scores (B) of male and female adolescent, young adult, and middle-aged test rats and no-shock controls. Negative cognitive bias scores > 0 are rats with a negative cognitive bias and scores ≤ 0 are rats that had a neutral or positive cognitive bias. Test rats have a greater negative cognitive bias than no-shock controls. In test rats, young adults and middle-aged rats had greater negative cognitive bias scores than adolescents, and males had greater negative cognitive bias than females in middle-age. *indicates *p*’s<0.000005: main effect of condition. *n*=6 for no-shock controls, *n*=8-11 for test rats. Figures modified and reprinted with permission from Hodges et al. [52]. **Table S1.** Correlations between inflammation and freezing in the ambiguous context or cognitive bias score. Bold * indicates *p*<0.05.  *n*=7-11 per group. **Table S2.** Correlations between doublecortin (DCX) in the dorsal and ventral hippocampus and freezing in the ambiguous context or cognitive bias score. Bold * indicates *p*<0.05. *n*=7-11 per group.** Table S3.** Correlations between DCX in the dorsal and ventral hippocampus and inflammation in the ventral hippocampus and basolateral amygdala. Bold * indicates *p*<0.05. *n*=7-11 per group.  
