# Supplementary material for: Sex differences in inflammation in the hippocampus and amygdala across the lifespan in rats: associations with cognitive bias
Source: Immun Ageing. 2022 Oct 6;19:43. doi: 10.1186/s12979-022-00299-4 (PMC9535862; doi:10.1186/s12979-022-00299-4)
Supplement: Supplementary file 1 — Additional file 1: Fig. S1. Heatmaps showing all correlations and cytoscape graphs showing significant correlations ≥0.7 between inflammatory marker correlations between the ventral hippocampus and basolateral amygdala in male and female adolescent (A-C), young adult (D-F), and middle-aged (G-I) rats after cognitive bias testing. The thickness of the lines in B, C, E, F, H, and I are related to the strength of the correlation (stronger is thicker), whereas the color relates to the valence (positive (red) or negative (blue)) of the correlation. Correlations of inflammatory marker levels within the ventral hippocampus and basolateral amygdala were largely positive in all age groups, although correlations between regions were more negative in male and female young adults compared to the other age groups. In adolescence, there was a sex difference in the correlations between basolateral amygdala IL-6 and cytokines (IFN-γ, IL-1β, IL-4, IL-5, IL-10, IL-13, TNF-α) in the ventral hippocampus, with positive correlations in adolescent females compared to negative correlations in adolescent males. n=7-11 per group. Fig. S2. Mean (±SEM) percentage of time spent freezing (A) and negative cognitive bias discrimination scores (B) of male and female adolescent, young adult, and middle-aged test rats and no-shock controls. Negative cognitive bias scores > 0 are rats with a negative cognitive bias and scores ≤ 0 are rats that had a neutral or positive cognitive bias. Test rats have a greater negative cognitive bias than no-shock controls. In test rats, young adults and middle-aged rats had greater negative cognitive bias scores than adolescents, and males had greater negative cognitive bias than females in middle-age. *indicates p’s<0.000005: main effect of condition. n=6 for no-shock controls, n=8-11 for test rats. Figures modified and reprinted with permission from Hodges et al. [52]. Table S1. Correlations between inflammation and freezing in the ambiguous context or cognitive bia [file 12979_2022_299_MOESM1_ESM.docx]

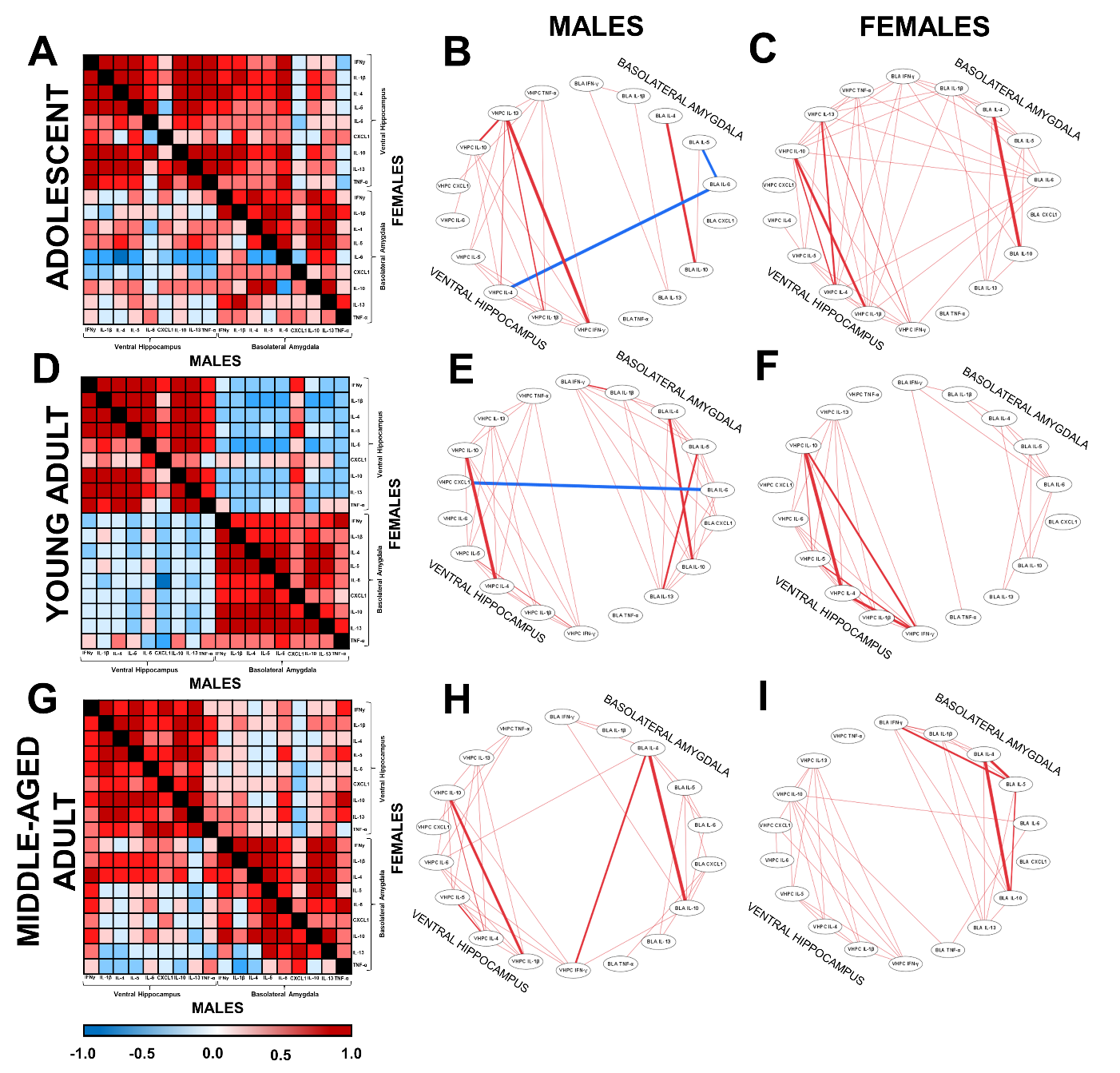


**Fig. S1.** Heatmaps showing all correlations and cytoscape graphs showing significant correlations ≥0.7 between inflammatory marker correlations between the ventral hippocampus and basolateral amygdala in male and female adolescent (**A-C**), young adult (**D-F**), and middle-aged (**G-I**) rats after cognitive bias testing. The thickness of the lines in **B**, **C**, **E**, **F**, **H**, and **I** are related to the strength of the correlation (stronger is thicker), whereas the color relates to the valence (positive (red) or negative (blue)) of the correlation. Correlations of inflammatory marker levels within the ventral hippocampus and basolateral amygdala were largely positive in all age groups, although correlations between regions were more negative in male and female young adults compared to the other age groups. In adolescence, there was a sex difference in the correlations between basolateral amygdala IL-6 and cytokines (IFN-γ, IL-1β, IL-4, IL-5, IL-10, IL-13, TNF-α) in the ventral hippocampus, with positive correlations in adolescent females compared to negative correlations in adolescent males. n=7-11 per group.

| **Table S1.** Correlations between inflammation and freezing in the ambiguous context or cognitive bias score. Bold * indicates p<0.05. n=7-11 per group. | | | | | | | | | | | | | | | | | | | | | | | | | | | | | | |
| --- | --- | --- | --- | --- | --- | --- | --- | --- | --- | --- | --- | --- | --- | --- | --- | --- | --- | --- | --- | --- | --- | --- | --- | --- | --- | --- | --- | --- | --- | --- |
|  | | Adolescent male | | | | | Adolescent female | | | | | Young adult male | | | | | Young adult female | | | | | Middle-age male | | | | | Middle-age female | | | |
|  | | Freezing | | Score | | Freezing | | | Score | | Freezing | | | Score | | Freezing | | | Score | | Freezing | | | Score | | Freezing | | | Score |  |
| Ventral  hippocampus | | | | | | | | | | | | | | | | | | | | | | | | | | | | | |  |
| IFN-γ | 0.174 | | 0.317 | | -0.237 | | | -0.034 | | -0.389 | | | -0.018 | | 0.137 | | | 0.099 | | 0.211 | | | 0.254 | | 0.003 | | | 0.193 | |  |
| IL-1β | 0.278 | | 0.021 | | 0.019 | | | 0.014 | | -0.576 | | | -0.153 | | 0.067 | | | 0.035 | | 0.094 | | | 0.357 | | -0.288 | | | 0.085 | |  |
| IL-4 | 0.145 | | -0.164 | | -0.006 | | | 0.077 | | -0.266 | | | 0.094 | | 0.134 | | | 0.105 | | -0.101 | | | 0.103 | | -0.133 | | | 0.005 | |  |
| IL-5 | 0.166 | | 0.208 | | -0.089 | | | 0.030 | | -0.446 | | | -0.113 | | 0.024 | | | -0.001 | | 0.062 | | | 0.124 | | -0.101 | | | 0.179 | |  |
| IL-6 | 0.008 | | -0.639 | | 0.207 | | | 0.334 | | -0.625 | | | -0.625 | | 0.143 | | | 0.092 | | 0.049 | | | 0.427 | | 0.095 | | | 0.376 | |  |
| CXCL1 | 0.542 | | 0.636 | | 0.138 | | | 0.201 | | -0.259 | | | -0.201 | | -0.388 | | | -0.425 | | -0.302 | | | 0.106 | | -0.222 | | | 0.012 | |  |
| IL-10 | 0.441 | | -0.331 | | 0.154 | | | 0.191 | | -0.236 | | | 0.084 | | 0.150 | | | 0.125 | | 0.071 | | | 0.269 | | 0.092 | | | 0.405 | |  |
| IL-13 | 0.373 | | 0.341 | | -0.237 | | | -0.166 | | -0.576 | | | -0.297 | | 0.037 | | | -0.014 | | 0.545 | | | 0.277 | | -0.032 | | | 0.273 | |  |
| TNF-α | 0.184 | | 0.537 | | -0.093 | | | 0.233 | | -0.163 | | | 0.104 | | 0.630 | | | 0.580 | | -0.556 | | | -0.053 | | -0.483 | | | -0.208 | |  |
| Basolateral  amygdala | | | | | | | | | | | | | | | | | | | | | | | | | | | | | |  |
| IFN-γ | -0.017 | | 0.424 | | 0.087 | | | -0.103 | | 0.251 | | | 0.339 | | 0.193 | | | 0.260 | | -0.433 | | | 0.154 | | -0.363 | | | -0.130 | |  |
| IL-1β | -0.140 | | -0.115 | | -0.038 | | | -0.138 | | 0.135 | | | 0.262 | | -0.101 | | | 0.011 | | -0.306 | | | 0.415 | | -0.347 | | | -0.013 | |  |
| IL-4 | 0.340 | | **0.851*** | | 0.456 | | | 0.250 | | 0.422 | | | 0.399 | | 0.184 | | | 0.237 | | -0.440 | | | 0.000 | | -0.366 | | | -0.121 | |  |
| IL-5 | 0.478 | | 0.422 | | 0.432 | | | 0.254 | | 0.133 | | | 0.125 | | 0.087 | | | 0.129 | | -0.012 | | | 0.165 | | -0.353 | | | -0.112 | |  |
| IL-6 | -0.394 | | -0.307 | | 0.281 | | | 0.220 | | 0.352 | | | -0.002 | | 0.131 | | | 0.176 | | -0.070 | | | -0.070 | | -0.052 | | | 0.424 | |  |
| CXCL1 | 0.453 | | 0.199 | | -0.382 | | | -0.197 | | 0.144 | | | -0.053 | | 0.084 | | | 0.145 | | -0.361 | | | -0.321 | | 0.429 | | | 0.474 | |  |
| IL-10 | 0.449 | | **0.719*** | | 0.545 | | | 0.269 | | 0.422 | | | 0.387 | | 0.156 | | | 0.209 | | -0.237 | | | 0.197 | | -0.422 | | | -0.128 | |  |
| IL-13 | -0.542 | | 0.057 | | 0.352 | | | 0.039 | | 0.280 | | | 0.233 | | 0.352 | | | 0.402 | | 0.416 | | | 0.296 | | -0.367 | | | 0.073 | |  |
| TNF-α | -0.165 | | 0.097 | | 0.042 | | | -0.256 | | 0.692 | | | 0.626 | | 0.512 | | | 0.588 | | 0.212 | | | -0.212 | | 0.165 | | | 0.557 | |  |

| **Table S2.** Correlations between doublecortin (DCX) in the dorsal and ventral hippocampus and freezing in the ambiguous context or cognitive bias score. Bold * indicates p<0.05. n=7-11 per group. | | | | | | | | | | | | | | | | | | | | | | | | | | | | | | |
| --- | --- | --- | --- | --- | --- | --- | --- | --- | --- | --- | --- | --- | --- | --- | --- | --- | --- | --- | --- | --- | --- | --- | --- | --- | --- | --- | --- | --- | --- | --- |
|  | | Adolescent male | | | | | Adolescent female | | | | | Young adult male | | | | | Young adult female | | | | | Middle-age male | | | | | Middle-age female | | | |
|  | | Freezing | | Score | | Freezing | | | Score | | Freezing | | | Score | | Freezing | | | Score | | Freezing | | | Score | | Freezing | | | Score |  |
| Dorsal DCX | 0.0128 | | -0.1326 | | 0.1117 | | | -0.2349 | | **-0.7872*** | | | **-0.7643*** | | -0.3776 | | | -0.4355 | | 0.2654 | | | -0.2744 | | -0.2008 | | | 0.2077 | |  |
| Ventral DCX | 0.2519 | | 0.2326 | | 0.1908 | | | 0.2651 | | -0.1128 | | | 0.0656 | | 0.0162 | | | -0.0600 | | 0.5771 | | | 0.1016 | | -0.3227 | | | -0.2652 | |  |

| **Table S3.** Correlations between DCX in the dorsal and ventral hippocampus and inflammation in the ventral hippocampus and basolateral amygdala. Bold * indicates p<0.05. n=7-11 per group. | | | | | | | | | | | | | | | | | | | | | | | | | | | | | | |
| --- | --- | --- | --- | --- | --- | --- | --- | --- | --- | --- | --- | --- | --- | --- | --- | --- | --- | --- | --- | --- | --- | --- | --- | --- | --- | --- | --- | --- | --- | --- |
|  | | Adolescent male | | | | | Adolescent female | | | | | Young adult male | | | | | Young adult female | | | | | Middle-age male | | | | | Middle-age female | | | |
|  | | Dorsal DCX | | Ventral DCX | | Dorsal DCX | | | Ventral DCX | | Dorsal DCX | | | Ventral DCX | | Dorsal DCX | | | Ventral DCX | | Dorsal DCX | | | Ventral DCX | | Dorsal DCX | | | Ventral DCX |  |
| Ventral  hippocampus | | | | | | | | | | | | | | | | | | | | | | | | | | | | | |  |
| IFN-γ | -0.4448 | | -0.1277 | | -0.0499 | | | -0.3264 | | 0.0294 | | | 0.0854 | | **0.728*** | | | 0.311 | | -0.211 | | | 0.280 | | 0.087 | | | 0.095 | |  |
| IL-1β | -0.3168 | | -0.1368 | | -0.0206 | | | -0.2551 | | 0.1196 | | | -0.0532 | | 0.629 | | | 0.112 | | -0.303 | | | 0.484 | | 0.158 | | | 0.375 | |  |
| IL-4 | -0.5127 | | 0.4639 | | -0.1772 | | | -0.4759 | | -0.1031 | | | 0.0302 | | 0.628 | | | 0.253 | | -0.231 | | | 0.520 | | -0.270 | | | 0.384 | |  |
| IL-5 | -0.4635 | | 0.1019 | | 0.0416 | | | -0.2856 | | 0.0922 | | | 0.071 | | **0.722*** | | | 0.175 | | -0.197 | | | 0.501 | | 0.181 | | | 0.240 | |  |
| IL-6 | -0.3374 | | -0.1661 | | -0.0075 | | | 0.1923 | | 0.1991 | | | -0.4893 | | 0.616 | | | 0.136 | | -0.261 | | | 0.496 | | 0.201 | | | -0.124 | |  |
| CXCL1 | 0.0053 | | -0.1271 | | -0.3196 | | | 0.4155 | | -0.2028 | | | -0.6869 | | **0.718*** | | | 0.235 | | -0.315 | | | 0.131 | | 0.203 | | | 0.096 | |  |
| IL-10 | -0.5334 | | 0.0636 | | -0.158 | | | -0.2711 | | -0.1199 | | | 0.0216 | | 0.636 | | | 0.226 | | -0.112 | | | 0.511 | | 0.109 | | | 0.088 | |  |
| IL-13 | -0.3578 | | -0.1004 | | -0.1261 | | | -0.4287 | | 0.1343 | | | -0.1272 | | **0.774*** | | | 0.277 | | -0.017 | | | **0.861*** | | 0.360 | | | 0.141 | |  |
| TNF-α | -0.1964 | | -0.1325 | | -0.3429 | | | -0.2221 | | 0.0649 | | | 0.4146 | | 0.264 | | | -0.138 | | -0.255 | | | **0.711*** | | -0.054 | | | 0.002 | |  |
| Basolateral  amygdala | | | | | | | | | | | | | | | | | | | | | | | | | | | | | |  |
| IFN-γ | -0.5949 | | 0.0161 | | -0.117 | | | -0.5909 | | -0.1108 | | | 0.188 | | -0.072 | | | -0.136 | | -0.409 | | | -0.517 | | 0.523 | | | 0.215 | |  |
| IL-1β | -0.6474 | | 0.0086 | | -0.3808 | | | -0.0435 | | -0.0931 | | | 0.0855 | | -0.080 | | | 0.133 | | **-0.722*** | | | -0.415 | | 0.481 | | | 0.222 | |  |
| IL-4 | -0.4165 | | 0.5422 | | -0.472 | | | -0.4223 | | -0.3332 | | | -0.0316 | | -0.175 | | | 0.126 | | -0.186 | | | -0.485 | | **0.616*** | | | 0.298 | |  |
| IL-5 | -0.5308 | | 0.5824 | | -0.5203 | | | -0.159 | | -0.0268 | | | 0.0943 | | -0.105 | | | -0.299 | | -0.257 | | | -0.318 | | **0.640*** | | | 0.208 | |  |
| IL-6 | 0.1155 | | -0.6178 | | -0.1095 | | | -0.3704 | | 0.0381 | | | 0.2966 | | -0.011 | | | -0.016 | | 0.212 | | | -0.236 | | 0.571 | | | 0.096 | |  |
| CXCL1 | -0.4334 | | 0.1844 | | -0.6629 | | | -0.2495 | | 0.1134 | | | 0.2037 | | 0.438 | | | -0.009 | | -0.132 | | | -0.061 | | 0.234 | | | -0.025 | |  |
| IL-10 | -0.5335 | | 0.5942 | | -0.3371 | | | -0.4236 | | -0.3611 | | | -0.0699 | | -0.128 | | | 0.113 | | -0.347 | | | -0.465 | | **0.665*** | | | 0.237 | |  |
| IL-13 | -0.6891 | | 0.1474 | | -0.4559 | | | -0.1606 | | -0.1027 | | | 0.1337 | | -0.356 | | | -0.552 | | 0.053 | | | -0.021 | | **0.690*** | | | 0.367 | |  |
| TNF-α | -0.391 | | -0.3833 | | -0.3182 | | | 0.2018 | | -0.5432 | | | 0.2759 | | -0.530 | | | -0.241 | | 0.246 | | | 0.581 | | 0.380 | | | -0.071 | |  |


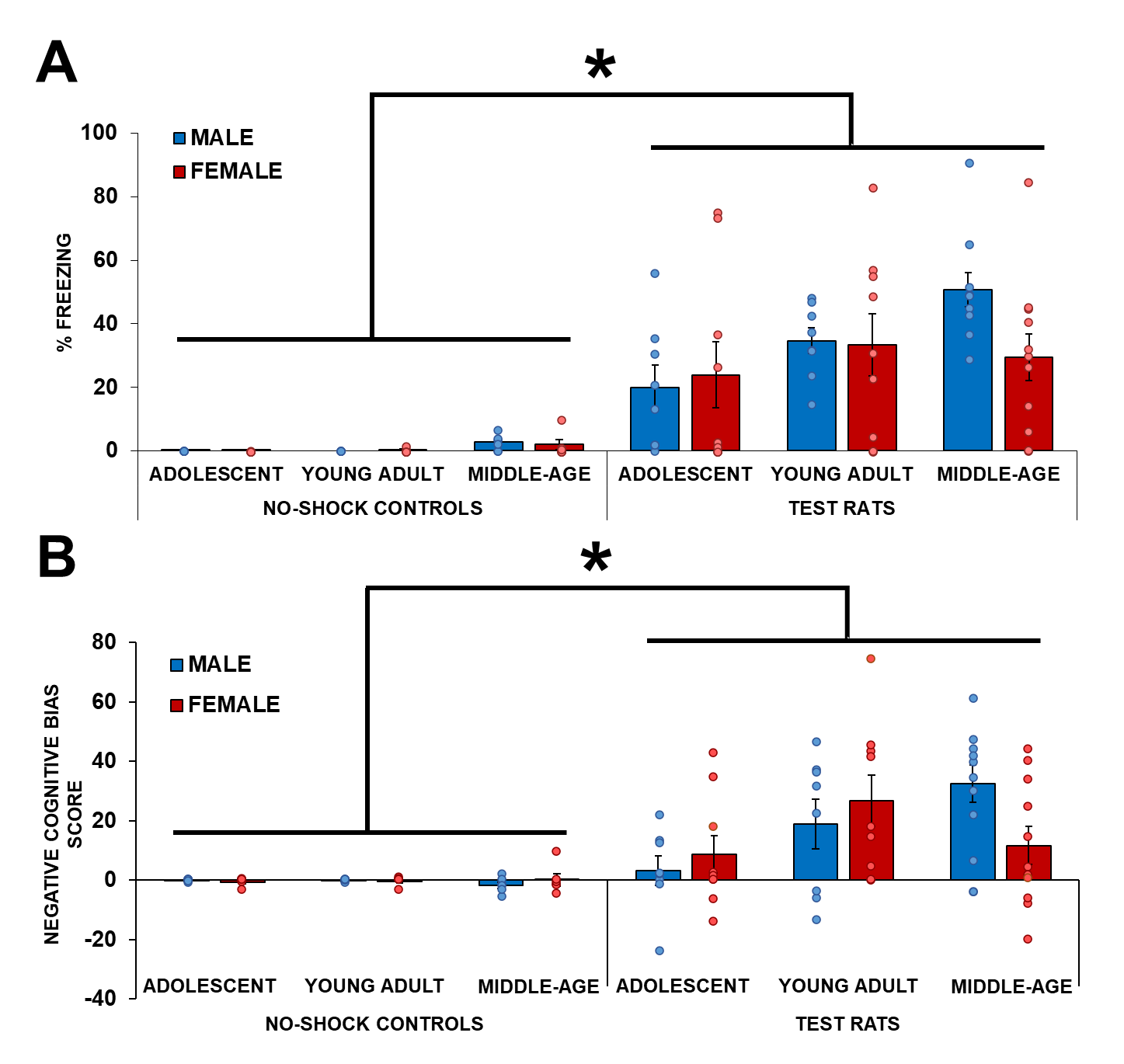


**Fig. S2.** Mean (±SEM) percentage of time spent freezing (**A**) and negative cognitive bias discrimination scores (**B**) of male and female adolescent, young adult, and middle-aged test rats and no-shock controls. Negative cognitive bias scores > 0 are rats with a negative cognitive bias and scores ≤ 0 are rats that had a neutral or positive cognitive bias. Test rats have a greater negative cognitive bias than no-shock controls. In test rats, young adults and middle-aged rats had greater negative cognitive bias scores than adolescents, and males had greater negative cognitive bias than females in middle-age. *indicates p’s<0.000005: main effect of condition. n=6 for no-shock controls, n=8-11 for test rats. Figures modified and reprinted with permission from Hodges et al. [52].
